# Characterization of individual stacking faults in a wurtzite GaAs nanowire by nanobeam X-ray diffraction

**DOI:** 10.1107/S1600577517009584

**Published:** 2017-08-09

**Authors:** Arman Davtyan, Sebastian Lehmann, Dominik Kriegner, Reza R. Zamani, Kimberly A. Dick, Danial Bahrami, Ali Al-Hassan, Steven J. Leake, Ullrich Pietsch, Václav Holý

**Affiliations:** a Faculty of Science and Engineering, University of Siegen, D-57068 Siegen, Germany; b Department of Solid State Physics/NanoLund, Lund University, Box 118, S-22100 Lund, Sweden; c Department of Condensed Matter Physics, Charles University, Ke Karlovu 5, 121 16 Praha, Czech Republic; d Center for Analysis and Synthesis, Lund University, Box 124, S-22100 Lund, Sweden; e ESRF – The European Synchrotron, 71 Avenue des Martyrs, 38000 Grenoble, France

**Keywords:** stacking faults, Patterson function, nanowire, coherent nanobeam X-ray diffraction

## Abstract

Coherent X-ray diffraction was used to measure the type, quantity and the relative distances between stacking faults along the growth direction of two individual wurtzite GaAs nanowires grown by metalorganic vapour epitaxy. The presented approach is based on the general property of the Patterson function, which is the autocorrelation of the electron density as well as the Fourier transformation of the diffracted intensity distribution of an object. Partial Patterson functions were extracted from the diffracted intensity measured along the  direction in the vicinity of the wurtzite  Bragg peak. The maxima of the Patterson function encode both the distances between the fault planes and the type of the fault planes with the sensitivity of a single atomic bilayer. The positions of the fault planes are deduced from the positions and shapes of the maxima of the Patterson function and they are in excellent agreement with the positions found with transmission electron microscopy of the same nanowire.

## Abstract 2

The application of the synchrotron-radiation-based coherent nanobeam X-ray diffraction method to study the type, quantity and the exact distances in between stacking faults in single GaAs nanowires is demonstrated.

## 1. Introduction   

GaAs nanowires (NWs), in contrast to GaAs bulk, can exhibit not only the stable zincblende (ZB) crystallographic phase with an *ABCABC…* bilayer stacking along the densely packed direction but also the metastable wurtzite (WZ) structure (McMahon & Nelmes, 2005 ▸) with periodic bilayer stacking of type *ABAB…*. It is even possible to tailor the NW structure from a ‘perfect’ defect-free single phase to alternating crystallographic phase NWs (Algra *et al.*, 2008 ▸; Caroff *et al.*, 2009 ▸; Joyce *et al.*, 2010 ▸; Lehmann *et al.*, 2013 ▸). The method of choice for investigating crystal structure and occurrence of stacking faults (SFs) in NWs is transmission electron microscopy (TEM) [see (Johansson *et al.*, 2006 ▸), for instance]; however, this method is destructive and the sample preparation is quite demanding. Semiconductor NWs have been investigated by X-ray diffraction [see a recent review by Stangl *et al.* (2013 ▸)]. The ‘standard’ laboratory X-ray diffraction (XRD) uses a partially coherent primary X-ray wave and it studies large ensembles of NWs (typically more than 10^6^) (Mandl *et al.*, 2006 ▸; Eymery *et al.*, 2007 ▸; Keplinger *et al.*, 2009 ▸; Köhl *et al.*, 2016 ▸); this approach yields average parameters of the NWs such as the facet orientation (Mariager *et al.*, 2007 ▸), size, chemical composition, crystal phases *etc*., which are relevant for optimizing the NW growth.

Single NWs can be investigated by the help of a very narrow and almost fully coherent primary X-ray beam (nanobeam). Coherent X-ray diffraction (CXD) utilizes the full coherence of a primary nanobeam and it has been used in several works for the determination of the shape of an individual NW (Diaz *et al.*, 2009 ▸; Chamard *et al.*, 2009 ▸; Biermanns *et al.*, 2009 ▸), as well as for the determination of the local crystal structure. In the paper by Favre-Nicolin *et al.* (2010 ▸) a reversed Monte Carlo fitting procedure has been used for the reconstruction of the ZB/WZ sequence, and a standard fitting method was used by Biermanns *et al.* (2012 ▸) for the determination of the lengths and positions of ZB and WZ segments. A direct phase-retrieval method was used by Davtyan *et al.* (2016 ▸) for the determination of the stacking fault density in a single NW. However, this work also demonstrates that the result of the phase-retrieval algorithm might be ambiguous when high defect densities are present.

Here, we aim to study the quantity, positions and the types of SFs along the growth direction in a single GaAs NW with a predominant WZ structure by performing an inverse Fourier transform from an experimentally measured speckle pattern. We show that the Fourier transform of the reciprocal-space distribution of diffracted intensity [the Patterson function (Patterson, 1935 ▸)] is a suitable method for a direct determination of the positions of the SFs in a chosen NW if the number of defects in the irradiated sample volume is sufficiently small, as illustrated in this work with up to five SFs. The method is much simpler than the approaches based on the retrieval of the phase of the diffracted wave mentioned above and has been recently demonstrated for GaN microcrystals (Holý *et al.*, 2017 ▸). Furthermore, the method is able to determine the distance between individual SFs with atomic precision and also identifies the types of defects, if not only the positions but also the shapes of the maxima of the Patterson function are considered.

The paper is structured as follows: §2 describes the sample preparation, characteristics and coherent X-ray diffraction setup. §3 introduces the basics of the theory of coherent diffraction from a NW with SFs. §4 describes the Patterson analysis and results from measured speckle patterns. Finally, in §5 we present the results of TEM which are correlated to the results obtained from partial Patterson analysis showing good agreement for the positions and type of the SFs in the NWs under investigation.

## 2. Sample preparation and X-ray diffraction experiments   

**Figure 1:**
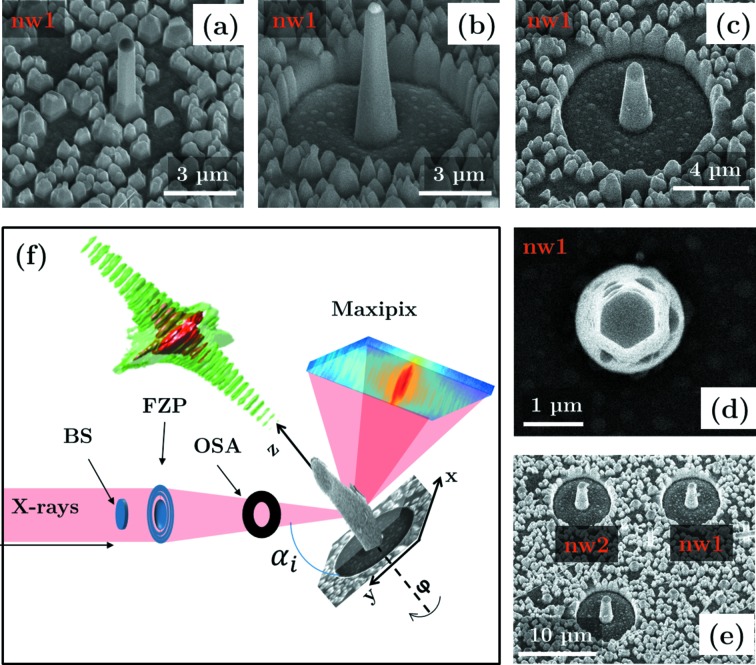
(*a*)–(*e*) SEM images of the focused ion beam sample preparation. (*a*) nw1 in the as-grown state. (*b*) nw1 after the FIB treatment to clean its surrounding from parasitic islands and after reducing its length (*c*). (*d*) Top view of the nanowires cross section after the final FIB treatment. (*e*, *f*) Overview images which show that multiple nanowires were prepared in a similar fashion. (*f*) Schematic of the experimental setup which shows the focusing Fresnel zone plate (FZP), beam stop (BS) and order-sorting aperture (OSA). The focused beam is almost fully coherent and when centred on the NW of interest leads to the observation of a speckle pattern. When several 2D images are combined to a 3D reciprocal-space map an intensity distribution as shown in the 3D contour plot is observed.

The GaAs NWs were grown by means of metalorganic vapour phase epitaxy (MOVPE) on epiready GaAs (111)B substrates in an AIXTRON 200/4 horizontal setup. Everywhere in this paper we represent the directions and planes in the hexagonal crystallographic base of the WZ lattice, *i.e.* we use four-digit Miller indices denoted *hkil* where the first three indices refer to the basal plane and the last index is along the growth axis. The growth conditions were optimized to achieved WZ GaAs NWs with a minimized number of stacking faults occurring along the 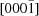-type growth direction. Details of the growth parameters can be found elsewhere (Lehmann *et al.*, 2012 ▸, 2013 ▸). Au particles with a density of about 18 µm^−2^ and diameter of 80 nm serve as catalytic seeds to control the density and the diameter of the NWs. A broad diameter distribution was observed directly after the growth; immersion of the sample in an ultrasonic bath (70/30 2-propanol/water mixture for 30 s) resulted in only relatively large diameters (*d* ≲ 200 nm) at a much lower density. Thus, an individual nanowire could be illuminated by an X-ray nanobeam. Investigation of the scanning electron microscopy (SEM) images [see Fig. 1(*a*) ▸] shows that a pyramidal base is formed at the bottom of every NW due to lateral overgrowth during the growth process which is a well known effect occurring under the conditions used (Lehmann *et al.*, 2012 ▸). Careful adjustment of the growth and processing parameters allowed us to control both the density and the crystallographic phase of the NWs. In our case the NWs exhibited mostly the WZ crystal structure [with the *ABAB…* stacking of the basal (0001) planes], with a low number of SFs.

As mentioned before, we observed pyramidal bases of NWs mostly being removed by the ultrasonic cleaning as a result of the crystal phase tuning. These residual bases (parasitic islands) can represent obstacles for CXD experiments. From the growth characteristics and preliminary investigations we know that the parasitic pyramids contain WZ segments and therefore scatter at the same Bragg condition as the NWs. In order to obtain an isolated NW, which is the necessary condition for CXD, we have employed an additional sample preparation step in a SEM equipped with a focused ion beam (FIB). In a circular area around a randomly chosen NW of interest we remove the parasitic islands by bombardment with Ga ions with an acceleration voltage of 30 keV and 0.92 nA beam current perpendicular to the sample surface. Although the chosen NW is not directly hit by the ion beam itself during this treatment, some material is deposited on the side walls of the NW. Comparison of the SEM images before [Fig. 1(*a*) ▸] and after the FIB cleaning [Fig. 1(*b*) ▸] shows that the deposited material is thicker in the bottom part (see also Fig. 9 for a TEM analysis of the redeposited material).

The necessary radius of this FIB cleaning step is dictated by the Bragg diffraction geometry of the CXD experiment. We have estimated that the radius of 4 µm is sufficient for a given length of the NW of around 2 µm, an X-ray energy of 8 keV, and the region of interest around the 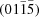 Bragg diffraction maximum, at around 60° angle of incidence. A further FIB processing step was used to cut the remaining Au-seed particle from the top of the NW. This step was necessary for two reasons: firstly to reduce the length of the NW [see Figs. 1(*b*) and 1(*c*) ▸] and secondly to be able to observe its internal cross section at the top. The top view after cutting the gold droplet [see Fig. 1(*d*) ▸] shows the hexagonal cross section of the NW surrounded by an amorphous shell created during the FIB treatment. The described FIB procedure was applied to several NWs. In this work we focus on the results of two such NWs labelled nw1 and nw2 in Fig. 1(*e*) ▸ (side view).

CXD experiments were carried out at beamline ID01 at the European Synchrotron (ESRF), employing the available nano-focusing setup (Chahine *et al.*, 2014 ▸). A sketch of the experimental geometry is presented in Fig. 1(*f*) ▸. The focusing of the primary X-ray beam was achieved by a 1800 nm-thick Au Fresnel zone plate (FZP) with a diameter of 300 µm and the width of the outermost Fresnel zone being 70 nm. The FZP was illuminated by a primary X-ray beam with an energy of 8 keV and placed ∼13.5 cm upstream of the sample. In addition, a beamstop and an order-sorting aperture (OSA) were used to suppress the transmitted direct beam and higher diffraction orders of the FZP, respectively. The resulting coherent beam has a size of approximately 250 nm × 300 nm full width at half-maximum (FWHM). As illustrated in Fig. 1(*f*) ▸, the beam size is smaller than the length of the NW and only a part of the NW is illuminated. Since the NW is composed predominantly of WZ structure, the angle of incidence and the detector plane have to be adjusted to fulfill the Bragg condition where only the WZ phase contributes to diffraction. This has been achieved firstly by choosing the correct angle of incidence and secondly by adjusting the angle of asymmetric atomic planes by rotating the NW around the growth axis; the chosen asymmetric planes occur with a period of 60°. By performing scans along the nanowire axis at different diffractometer angles, how­ever, we have verified that, for a sufficiently small range (around 2°), an angular scan does not result in a significant change of the illuminated area on the NW. Once the Bragg diffraction is found, the typical speckle pattern around the WZ diffraction maximum is recorded by a two-dimensional (2D) MaxiPix detector with 516 × 516 pixels of 55 µm × 55 µm size mounted at a distance of 728 mm from the sample. The speckle pattern is the result of interference between the WZ segments separated by SFs (Favre-Nicolin *et al.*, 2010 ▸; Davtyan *et al.*, 2016 ▸). A three-dimensional (3D) reciprocal-space map of the diffracted intensity can be recorded by combining a series of 2D diffraction patterns collected at different angles of incidence. At the same time we also change the angle of the detector arm to move approximately along the [0001] crystal truncation rod, where the speckle pattern of the defects can be seen. For conversion from angular to reciprocal space we use the approach proposed by Kriegner *et al.* (2013 ▸). Using the scanning technique described above it is also possible to measure the asymmetric diffraction of other crystallographic phases that may occur in the GaAs nanowires, namely ZB with the bilayer stacking *ABCABC…*, and the twinned zinc-blende lattice (TZB) with the bilayer stacking *ACBACB…*.

**Figure 2:**
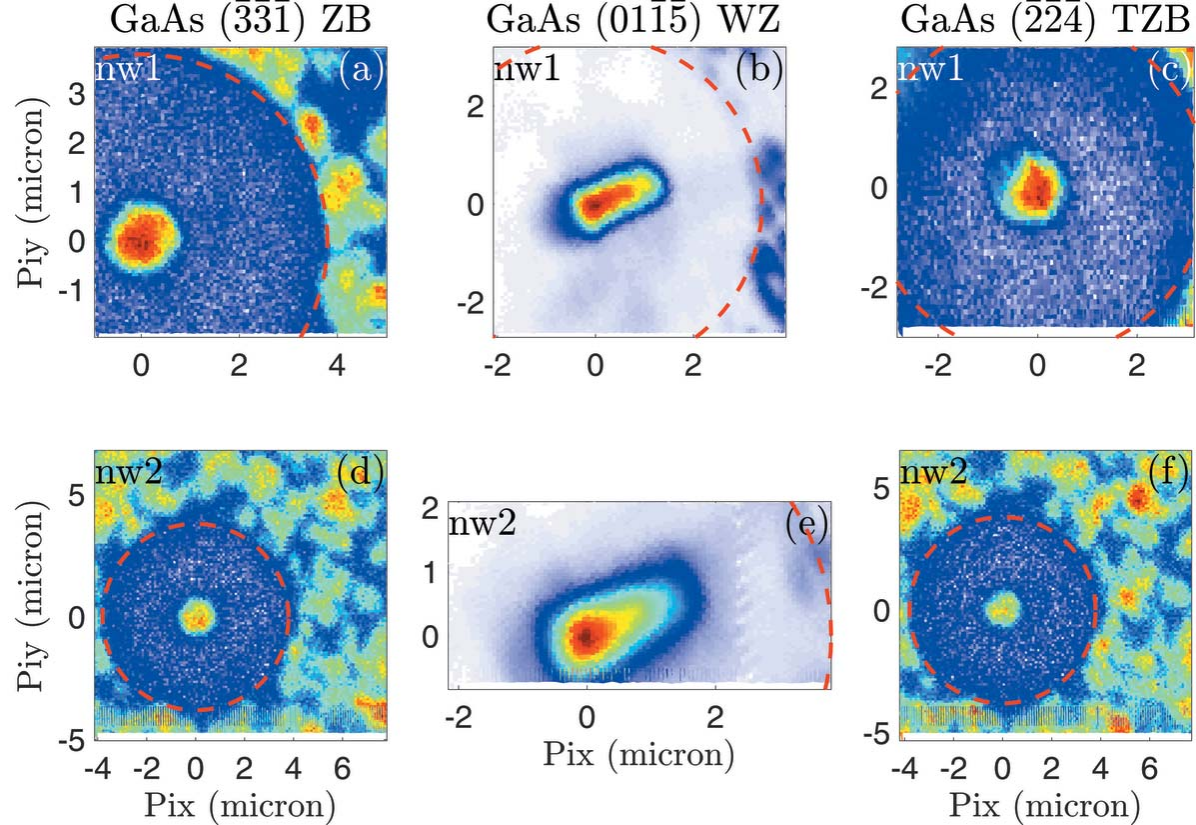
Scanning X-ray diffraction images of nw1 and nw2 and their surrounding for various Bragg peaks are shown. Images created by the GaAs ZB 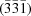, WZ 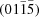 and twinned ZB 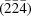 Bragg peaks are shown in panels (*a*, *d*), (*b*, *e*) and (*c*, *f*), respectively. The colour scale is adjusted to yield an appropriate contrast within the image. Especially at the 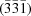 peak the constant contribution of the substrate is ignored. The NW appears as an elongated object since the incidence angles at the Bragg conditions are between 59 and 62°.

In order to reveal the crystal structure of the NWs and parasitic islands, we employed scanning X-ray diffraction, in which the sample is scanned across the primary nanobeam. The scanning was performed in three diffraction maxima of the ZB, WZ and TZB lattices. The resulting real-space crystallographic phase maps for nw1 and nw2 are presented in Fig. 2 ▸. It is obvious that the parasitic islands mostly consist of ZB and TZB segments, in contrast to wurtzite NWs. Note that the base of the NW of interest is also a parasitic island, since it is depicted by intensity maxima in the ZB and TZB images [see Figs. 2(*a*), 2(*c*) ▸ and 9(*c*) for nw1 and Figs. 2(*d*) and 2(*f*) ▸ for nw2]. On the other hand the NW itself consists only of WZ since its elongated shape shows up only in Figs. 2(*b*) and 2(*e*) ▸. It is important to note that very small ZB segments (containing one or a few *ABCABC…* stacked basal layers) cannot be detected by this technique. The real-space maps also show clearly the effect of FIB isolation by means of a clean, less intense, circle around the NW where only diffraction signal from the substrate can be detected.

**Figure 3:**
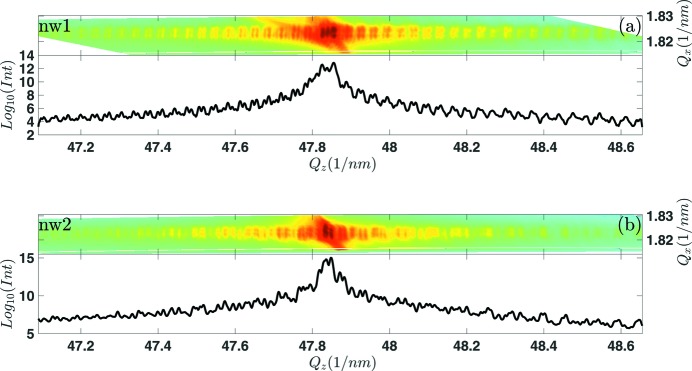
2D RSM of nw1 (*a*) and nw2 (*b*) with corresponding line cut at particular 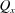 value.

The 3D reciprocal-space maps (RSMs) from nw1 and nw2 are measured at specific regions along the NW growth axis by performing the angular scan along the crystal truncation rod as described in the previous section around the 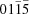 WZ diffraction. The projections of these maps onto the 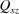 plane, where 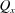 is the reciprocal-space axis parallel to the sample surface and to the azimuthal direction of the primary beam, and 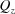 is perpendicular to the surface, are shown in Fig. 3 ▸. Line cuts from these projections along 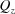 shown in Fig. 3 ▸ are used in the partial Patterson analysis described below. It is evident from the figure that nw1 shows more ordered and simple oscillations along 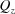 with about three periods superimposed, while the structure of the oscillations of nw2 is more complex. This implies that fewer defects occur in the illuminated part of nw1 than in nw2, since NWs with many defects do not exhibit a clear periodicity in the speckle patterns (Davtyan *et al.*, 2016 ▸).

## 3. Diffraction simulations   

In order to describe the speckle pattern arising from stacking defects along the 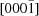 direction as shown in Fig. 3 ▸ we developed a simple theoretical description. Within the kinematical approximation and the far-field limit, the amplitude of the wave diffracted from a single NW is proportional to the Fourier transformation of its electron density. If we restrict to a distribution of the diffracted signal along the 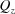 direction, *i.e.* parallel to the NW axis [0001], the expression for the amplitude of the scattered radiation has the following simple form,
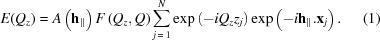
Here we have denoted 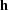 the reciprocal lattice vector, 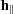 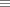 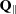 is the component of 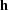 parallel to the sample surface (*i.e.* perpendicular to the NW axis), 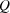 = 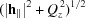 is the length of the scattering vector, and
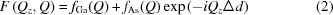
is the structure factor of a GaAs molecule. In the last expression, 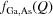 are the atomic form factors of Ga and As, respectively, and 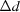 is the Ga—As bond length. In equation (1) we describe the NW structure as a sequence of basal (0001) planes, and the shape of the NW cross section is included in the pre-factor 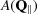; its particular form is not considered here. *N* is the total number of basal planes in the coherently irradiated part of the NW, 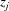 is the *z*-coordinate of the *j*th plane and 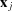 is the vector of its lateral shift with respect to the first basal plane.

In a perfect WZ structure, the basal planes are equidistantly distributed, *i.e.* 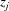 = 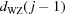, where 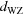 = 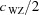 is the distance of basal (0001) planes and 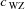 is the vertical WZ lattice parameter. In the case of a purely geometrical ZB 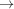 WZ transformation, this lattice parameter is connected with the cubic lattice parameter 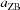 by the obvious formula 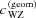 = 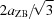; however, the actual lattice parameter differs by 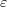 = 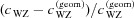 ≃ 1% (McMahon & Nelmes, 2005 ▸; Jacobsson *et al.*, 2015 ▸). Since the WZ structure exhibits the *ABAB…* bilayer stacking of the basal planes, the lateral shifts of the basal planes are 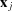 = 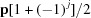, *i.e.* 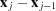 = 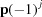. Here we denoted 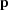 = 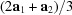, with 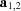 the basis vectors in the basal (0001) plane. The vectors 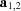 have a length of 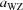 and the angle between them is 120°. In the case of the ZB lattice with the bilayer stacking *ABCABC…*, the lateral shifts are 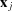 = 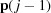, *i.e.* 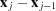 = 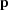.

**Figure 4:**
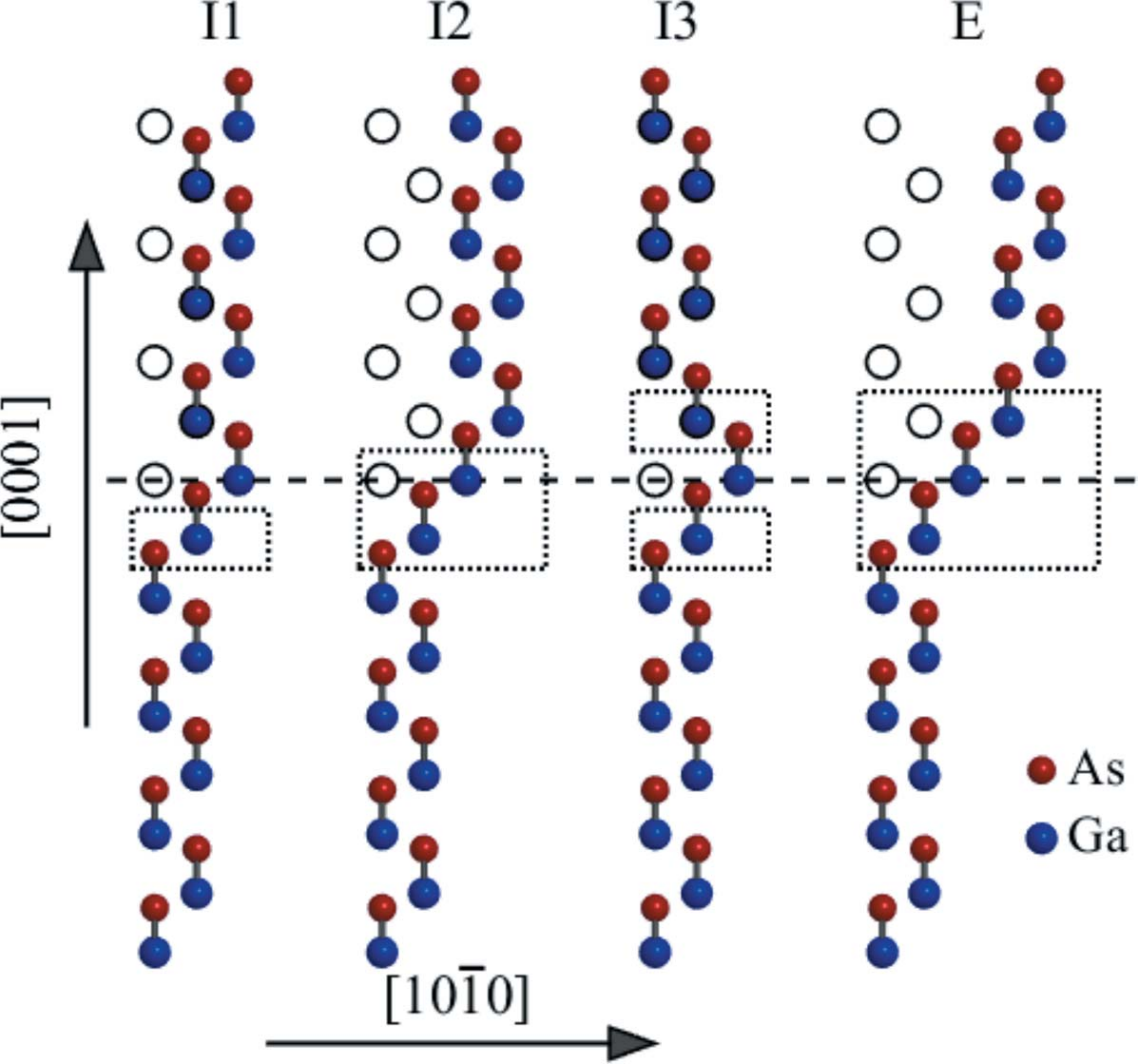
Types of SFs in wurtzite lattice. The horizontal dashed line represents the position of the fault plane, and the dotted rectangles denote small face-centred-cubic-like segments with *ABCABC…* stacking.

A real NW structure exhibits a random sequence of SFs. In a wurtzite lattice four types of SFs (Zakharov *et al.*, 2005 ▸), denoted I1, I2, I3 (intrinsic) and E (extrinsic), exist (see Fig. 4 ▸). In this sketch the position of the fault plane is denoted by the horizontal dashed line, and the empty circles represent the Ga positions in the ideal lattice. The dotted rectangles denote the ZB segments associated with a given SF; various fault types differ in the lengths of the ZB segments. We assume that the basal plane distance in the ZB segments corresponds to the ideal ZB lattice, *i.e.* 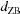 = 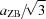. Outside the ZB segments we use the actual WZ inter-planar distance 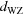 including the factor ∊. In the simulation we choose the positions 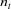, 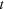 = 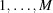, and types of individual SFs. Here we denote *M* the number of the SFs in the irradiated volume of the NW, and the numbers 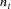 are the indices of the basal (0001) planes containing the faults. Then we generate the sequence of the *z*-coordinates 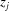, 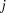 = 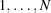, of the basal planes and the sequence of their lateral positions 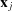.

In order to account for a limited coherence of the primary X-ray beam, the scattered intensity has to be calculated by the formula
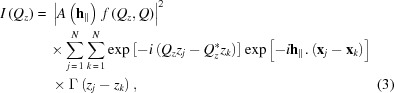
where 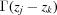 is the effective mutual coherence function of the experimental setup, containing both the coherence function of the primary radiation and the angular resolution of the detector (see below). This simulation, however, is time-consuming, since it requires summation of 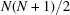 terms for each value of 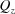. In order to speed up the procedure, we calculated the diffracted intensity using 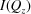 = 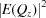 and equations (1) and (2) for the diffracted amplitude, and convoluted the resulting intensity distribution by the Fourier transformation of 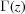. We have tested numerically that both approaches yield nearly identical results. A Gaussian function with a FWHM of 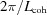 was used as the Fourier transformation of Γ; 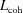 is the corresponding effective coherence length which depends upon the coherence properties of the primary beam and on a finite angular resolution of the 2D detector. In our experimental setup the primary beam was fully coherent across the beam diameter so that its coherence width 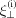 was large and its degree of coherence was determined only by its coherence length (Born & Wolf, 1999 ▸; Leake *et al.*, 2009 ▸),
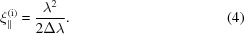
Here, λ is the wavelength and 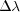 is the width of the wavelength spread determined by the primary monochromator. The effective coherence length of the primary beam along the *z*-axis (*i.e.* along the NW) is therefore 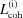 = 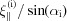, where 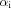 is the incidence angle of the primary radiation. The finite resolution of the detector can be described by the effective coherence width,
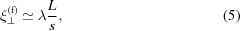
where *L* is the sample–detector distance and *s* is the size of the detector pixel; this formula follows from the optical reciprocity theorem (Born & Wolf, 1999 ▸). The coherence width determines the effective coherence length of the emitted beam along the *z*-axis: 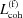 = 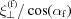, where 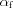 is the angle of exit. Combining both effects we obtain the effective coherence length along the *z*-axis used in the simulations,
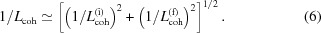
In our experimental setup we used 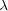 ≃ 1.5 Å, 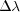 ≃ 10^−4^ Å, 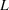 ≃ 0.7 m, 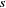 ≃ 0.05 mm, 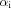 ≃ 60° and 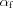 ≃ 20°, so that 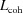 ≃ 1.3 µm. In our simple one-dimensional simulation we did not include the actual shape of the primary wavefront, since it would affect only the intensity profile across the 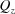-rod, which is not considered here.

**Figure 5:**
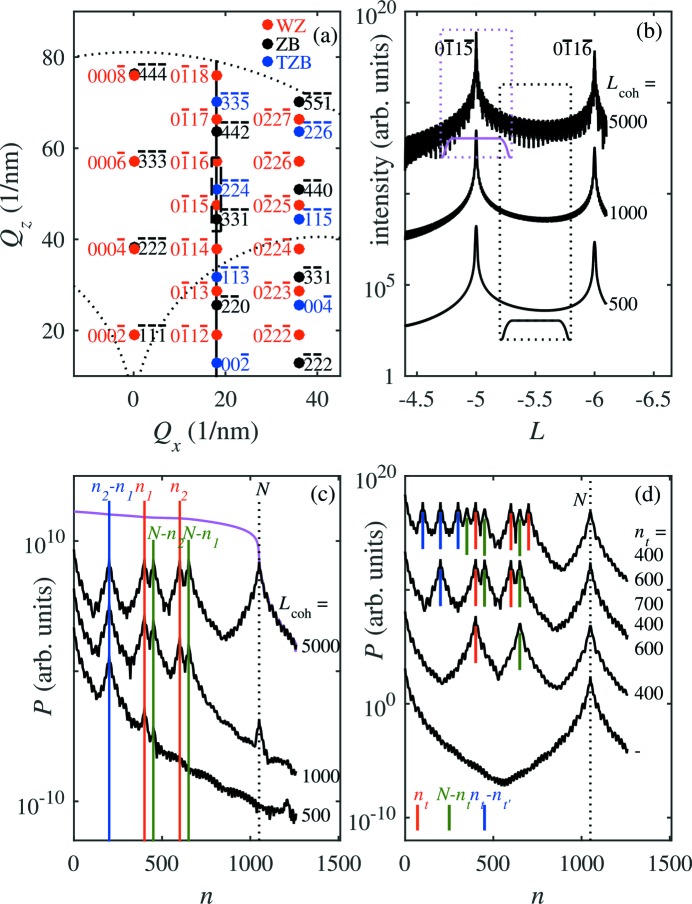
(*a*) Reciprocal-lattice points of ZB (black), WZ (red) and twinned ZB lattices (blue). The vertical black line denotes the trajectory of the 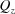-scans considered in this paper, and the dashed rectangle corresponds to the 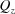-range used in panel (*b*). (*b*) The diffraction curve simulated for a pair of I1-type stacking faults in positions 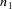 = 400 and 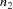 = 600 and using various effective coherence widths 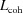. The parameters of the curves are the coherence widths expressed as multiples of the distances 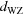 of the (0001) basal planes. The black and magenta rectangles denote the 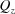-windows used for the calculation of the Patterson function; the shape of the Planck-taper window function is indicated by the small graphs in the rectangles. (*c*) The Patterson functions calculated from the diffraction curves in panel (*b*); the black and magenta curves were obtained using the black and magenta 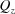-windows in (*b*), respectively. The vertical blue, red and green lines denote the maxima of the Patterson functions; see the main text for details. (*d*) The Patterson functions calculated for zero, one, two and three stacking faults of type I1; the positions 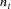 of the SFs are the parameters of the curves. In the calculation the coherence width 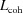 = 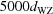 was used. In panels (*c*) and (*d*) the vertical dotted line *N* denotes the peak corresponding to the finite length of the nanowire. The curves in panels (*b*) to (*d*) are shifted vertically.

In the following we show several examples of the diffracted intensities simulated along the line in reciprocal space parallel to [0001] and crossing the reciprocal points 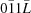 of the WZ lattice; the simulation line is sketched in Fig. 5*(a*) ▸. This line contains both the reciprocal points 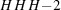 of the ZB lattice and points 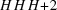 of the TZB lattice. Fig. 5(*b*) ▸ shows the diffraction curves simulated for two SFs of type I1 lying in positions 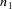 = 400, 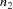 = 600 calculated for various coherence widths 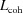 (in the figure expressed as multiples of the inter-planar distance 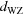). The total irradiated NW length was set to 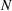 = 1050. The curve exhibits the WZ maxima with 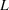 = 5, 6 and plenty of oscillations between them.

In order to analyze the diffraction curve, we calculate the partial Patterson function, *i.e.* the Fourier transformation of the diffracted intensity,
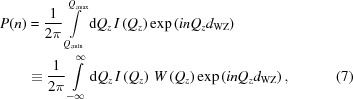
in a limited window 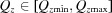. In order to suppress non-physical oscillations caused by the edges of the window function, we used a smoothing window function 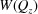 of the Planck-taper type (Tu, 2007 ▸), which is smooth everywhere (the class 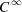 function) and exactly zero outside the region 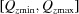.

Fig. 5(*c*) ▸ presents the Patterson functions calculated in two windows denoted in Fig. 5(*b*) ▸ by black and magenta rectangles; the shape of the window function is indicated by black and magenta curves in the rectangles. It is obvious that the Patterson function obtained from the window containing the main diffraction maximum [the magenta curve in Fig. 5(*c*) ▸] exhibits only the main maximum at 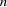 = 0. However, the Patterson function calculated from the window *between* the maxima [the black rectangle in Fig. 5(*b*) ▸ and the black curves in Fig. 5(*c*) ▸] shows several sharp maxima. The maxima in the curve obtained for large 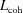 correspond to the positions 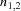 of the SFs [the red vertical lines in Fig. 5(*b*) ▸], the ‘mirror’ positions 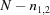 (the green lines) and the difference 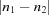 of the positions (the blue line). An additional maximum appears at 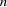 = 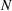, which corresponds to the full irradiated NW length (the black dotted line). Decreasing the coherence width, the maxima on the Patterson function gradually disappear. If 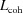 < 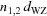 and 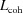 < 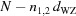, only the difference peak at 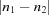 persists; this is the case for the curve calculated for 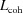 = 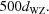 In Fig. 5(*d*) ▸ we display the Patterson functions calculated for a large coherence width 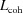 = 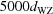 and various numbers of SFs. If the coherence width is large, the analysis of the Patterson function is complicated due to the presence of various types of peaks (denoted by various colours in the figure). In this case, a suitable low-pass filter can be applied before the Fourier transformation.

**Figure 6:**
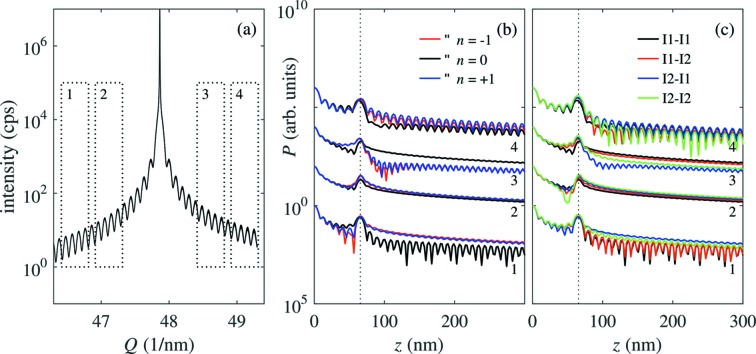
(*a*) Diffraction curve simulated for a pair of SFs, using the effective coherence width of 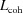 = 2 µm, and the SF positions 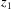 and 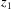 + 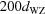 = 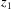 + 65.6 nm; the rectangles represent the 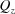-windows used for the simulation of the Patterson functions in panels (*b*) and (*c*). (*b*) The Patterson functions calculated for various distances 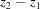 = 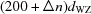 of the I1–I1 SF pair. The parameters of the curves correspond to the numbers of the 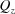-windows in panel (*a*); the colours of the curves denote the values of 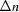. (*c*) The Patterson functions calculated for the same SF positions but different SF types; the types of the SFs correspond to different colours of the curves. The curves corresponding to different 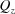-windows are shifted vertically.

Performing the Patterson analysis of an intensity curve 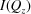 for various positions of the window function, from the *shape* of the Patterson function it is possible to determine the type of the SFs and their exact positions, the latter with much better accuracy than from the positions of the peaks in 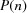. This fact is demonstrated in Fig. 6 ▸, where we plot the diffraction curve with various 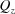-windows [Fig. 6(*a*) ▸], and the corresponding Patterson functions [Figs. 6(*b*) and 6(*c*) ▸]. The 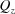-windows exclude the region of the main Bragg peak and their widths correspond to the 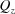-range used in the analysis of the experimental data.

The spatial resolution of the Patterson function depends on the length of the chosen 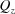-window. In Fig. 6(*b*) ▸ we considered a pair of SFs of type I1, having various distances of the fault planes differing by a single basal (0001) plane [Fig. 6(*b*) ▸]; in Fig. 6(*c*) ▸ we show the Patterson function for various combinations of types I1 and I2 and the same SF distance. Other fault types are not shown since they appear with much lower probability and were not observed in our experiments as will be shown further below. Calculating these curves, we used the actual value of 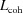 = 2 µm and the 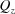-windows used in the analysis of the experimental data in the next section. From the figure it is obvious that the change of the distance of the SFs by ±1 basal plane almost does not move the maximum of the Patterson function, but it affects substantially the *shape* of the tails of the maxima. Similar effects can be observed for various types of SFs. The maxima of the Patterson functions obtained from different 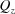-windows exhibit different shapes, characteristic both of the exact SF distance and of the SF types, so that from the Patterson functions from various windows it is possible *in principle* to determine the exact distance of the SFs *and* their types.

## 4. Patterson analysis of the experimental data   

**Figure 7:**
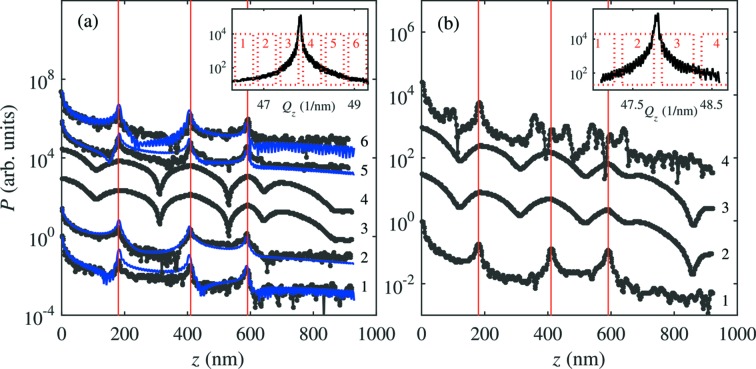
The Patterson functions calculated from the 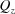 scans extracted from two independent 3D reciprocal-space maps [panels (*a*) and (*b*)] of nw1 depicted in the SEM images in Fig. 1 ▸. The insets show the 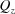 scans along with the positions of the 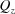-windows from which the Patterson functions have been obtained. The main panels display the Patterson functions; the parameters of the curves refer to the 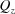-windows, the curves are shifted vertically for clarity. The vertical red lines mark the maxima of the Patterson functions; it is obvious that the functions calculated from different 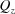-windows and the functions obtained from various scans in panels (*a*) and (*b*) exhibit the maxima in the same positions. The Patterson functions from the scan in panel (*a*) have been fitted to the model described in the main text; the fitted curves are denoted by blue lines.

From the line scans in Fig. 3 ▸ we calculated the partial Patterson functions using various 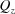-windows. Figs. 7 ▸, for nw1, and 8 ▸, for nw2, show the 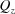 scans along with the 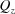-windows and the corresponding partial Patterson functions. The Patterson functions of nw1 show three distinct maxima; their positions are identical in the Patterson functions calculated from various 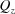-windows and also in Patterson functions from two independent measurements [Figs. 7(*a*) and 7(*b*) ▸]. The positions of the maxima are at 183 nm, 415 nm and 598 nm with an accuracy of about ±10 nm. If we assume that the coherently irradiated part of the NW does not contain the ends of the NW, or the end of the NW is not very well defined due to the FIB cutting of the top, then these maxima correspond only to the distances between the SFs, analogously to the blue lines in Figs. 5(*c*) and 5(*d*) ▸. From the positions of the maxima of the Patterson function we obtained unambiguously the coordinates of the SFs: 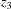, 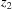 = 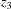 + 183 nm and 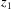 = 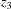 + 598 nm, where the coordinate of the last SF 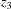 cannot be determined from the Patterson function.

The quality of the measured scan of nw1, shown in Fig. 7(*a*) ▸, made it possible to determine the exact distance of the SFs. To improve the robustness of this determination we used the types of the SFs determined from high-resolution TEM (HRTEM) images shown in the next section. From this analysis it follows that the types of the SFs are I1 in positions 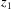 and 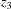, and I2 in position 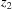. Using this *a priori* information, we fitted the experimental Patterson functions from various 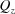-windows with the simulations; the results are shown in Fig. 7(*a*) ▸. From the figure it follows that the simulated curves agree qualitatively well with the measured data. The fit yielded the SF positions 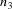, 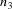 + 555 and 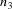 + 1793, which corresponds to the SF coordinates 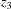, 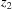 = 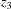 + 182.1 nm and 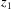 = 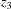 + 588.4 nm, respectively; these values are determined with an accuracy of 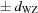 ≃ 0.3 nm.

**Figure 8:**
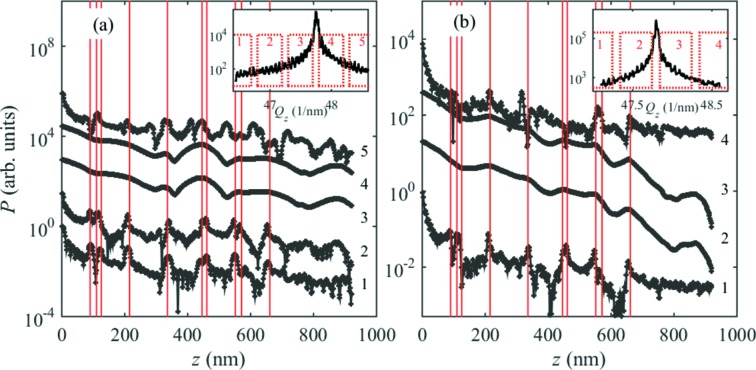
The same situation as in Fig. 7 ▸; nw2. The quality of the experimental Patterson function does not allow for fitting to the theoretical model.

In contrast to nw1, the Patterson function of nw2 (Fig. 8 ▸) is more complicated and it does not allow for a straightforward determination of the positions of the SFs. Nevertheless, analagous to nw1, the distinct maxima of the Patterson functions obtained from various 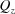-windows are at the same positions.

## 5. Transmission electron microscopy   

**Figure 9:**
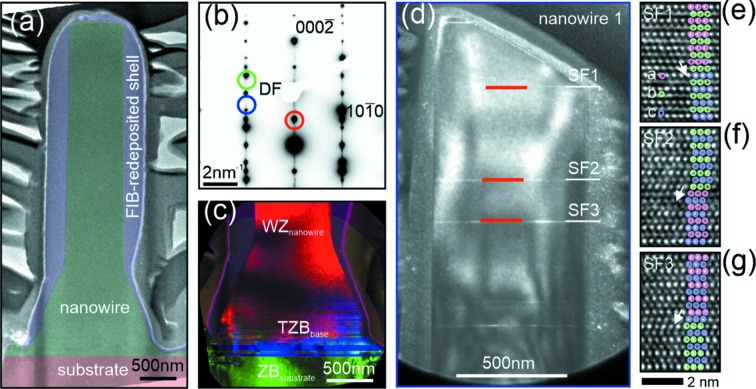
(*a*) Bright TEM overview image of an exemplary GaAs NW lamella with the substrate, NW body and FIB-redeposited shell post coloured for better visibility. A selected area diffraction pattern of the entire NW (*b*) and a convoluted dark-field image of the the base (*c*) using the diffraction spots for imaging as indicated in (*b*) show the mixed crystal structure at the base. A dark-field image of nw1 is given in (*d*) using the diffraction spot marked blue in (*b*) showing the stacking defects as brighter contrast lines in the otherwise darker WZ nanowire body. HRTEM details of the three stacking defects in the area of interest are given in (*e*)–(*g*) with coloured circles indicating the different bilayer stacking order.

In order to correlate the results of the Patterson analysis above with the structural properties of the NWs directly, we performed a detailed TEM investigation of the selected NWs using bright-field and conventional dark-field imaging as well as energy-dispersive X-ray spectroscopy. Fig. 9 ▸ shows a bright-field image of an exemplary nanowire FIB lamella with the substrate, NW body and FIB-redeposited shell post coloured for better visibility. The FIB-redeposited shell was determined to be amorphous with a compositional ratio of approximately 61% Ga and 39% As. A selected area diffraction pattern of the entire NW is displayed in Fig. 9(*b*) ▸ highlighting the diffraction spots chosen for the conventional dark-field imaging for crystal phase distinction in larger field-of-view images [for a more detailed experimental description of the approach on similar samples, see Lehmann *et al.* (2015 ▸)]. The base of the NW consists of WZ, ZB and twinned ZB segments, as can be seen in Fig. 9(*c*) ▸ by convoluting the crystal phase-sensitive dark-field images of all three phases. This supports the finding of CXD as discussed above. A dark-field TEM image of nw1 taken by choosing a ZB-sensitive diffraction spot shows the stacking defects as brighter contrast lines perpendicular to the NW axis (bottom to top) in the otherwise darker contrast WZ body of the nanowire. Due to strain in the lamella, additional contrast variations occur within the nanowire body. Detailed HRTEM images taken at the positions of the three different stacking defects are given in Figs. 9(*e*)–9(*g*) ▸. Coloured circles are additionally shown for better visibility of the different bilayer stacking sequences. The three SFs labelled are actually the ones probed by the aforementioned Patterson analysis.

From this analysis we obtained the distances between the SFs 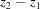 and 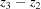; of course, the absolute position 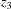 cannot be determined. We compared these distances with the positions of the SFs in Fig. 9(*d*) ▸ and we achieved an excellent agreement. Based on the HRTEM data presented in Figs. 9(*e*)–9(*g*) ▸ we can extract the type of the SFs. As explained in §3, the I1-type SF contains a ZB-like block of basal planes containing one single ZB-like plane, while the ZB block in I2 contains two ZB-like planes. Therefore, the types of the SFs in nw1 are I1, I2 and I1.

**Figure 10:**
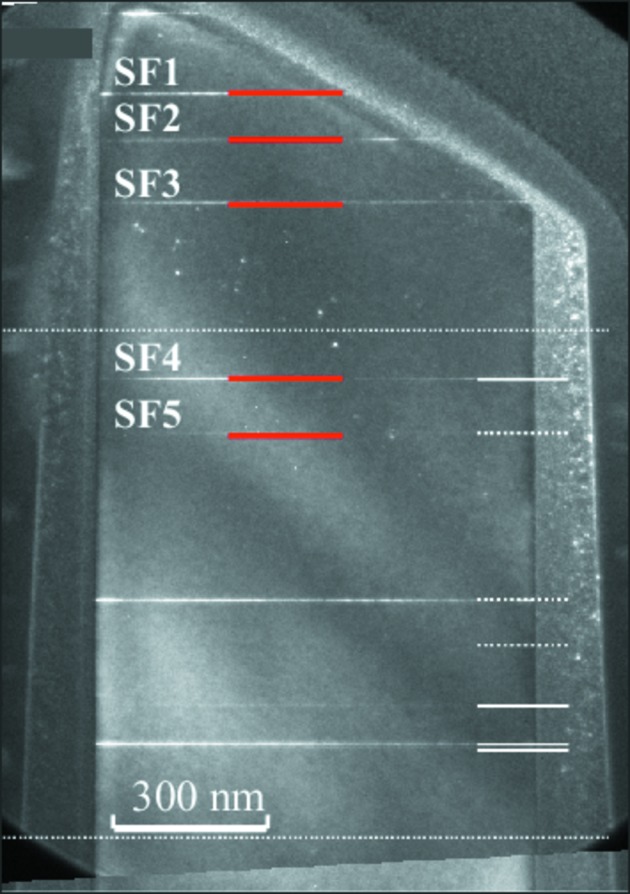
Cross-section TEM image of nw2. The horizontal dotted lines roughly denote the irradiated area; individual SFs are highlighted by horizontal white lines. The red lines mark the SFs used for the calculation of the positions of the maxima of the Patterson function; the maxima are shown in Fig. 8 ▸ as vertical red lines.

The cross-sectional conventional dark-field TEM image of nw2 in Fig. 10 ▸ shows that the NW contains more SFs than nw1. Nevertheless, for five SFs denoted by horizontal red lines in Fig. 10 ▸ the corresponding positions of the maxima of the Patterson function correspond very well to the experimental data (vertical red lines in Fig. 9 ▸).

## 6. Discussion   

The Patterson functions calculated from simulated 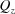 intensity distributions revealed that the appearance of the maxima on the Patterson function strongly depends on the coherence properties of the primary beam and/or on the angular resolution of the detector [see Figs. 5(*b*) and 5(*c*) ▸]. In the theoretical part of this paper we combined both these factors into one effective coherence length 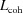 and we showed that, if this length is sufficiently large, several *types* of the maxima appear, corresponding to the absolute positions 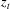 of the SFs, the mirror positions 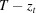 (*T* is the total length of the irradiated NW), as well as to the distances 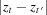. In this ideal case the interpretation of the Patterson function is complicated. If 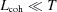, only the difference peaks 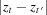 occur, which allows for a direct determination of 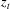, relative to the first or last SF in the NW. Moreover, as demonstrated above for nw1, even if the top part of the NW is influenced by FIB cutting, it is still possible to obtain the SF distances. In the nanowire nw1, with three SFs and given coherence properties of the beam, we have shown for two independent measurements that the Patterson function encodes the number of SFs and the relative distance between them. As has been demonstrated in §3, the *shape* of the Patterson function strongly depends on the distance between the SFs with a sensitivity of ±1 atomic plane. With the current resolution of the experimental speckle pattern it is difficult to achieve such a sensitivity only from the *positions* of the Patterson function maxima. The sensitivity of the *positions* of the maxima of the Patterson function to the SF positions can be estimated from the sampling theorem by
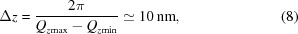

*i.e.* it is inversely proportional to the width of the 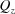-window. The sensitivity of the method to the SF distances can be substantially improved if we consider not only the positions of the maxima of the Patterson function but also its shape. Fig. 6(*b*) ▸ demonstrates that the shape of the maximum of the Patterson function changes if the SF distances change by *one atomic plane*, *i.e.* by ±0.3 nm [see Fig. 6(*b*) ▸]. In addition, the reliability of the distance determination improves if we take into account the Patterson functions obtained from several 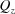-windows on the same 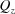 scan.

However, the single-plane sensitivity and the determination of the types of the SFs are possible only from experimental data with excellent quality and low noise. In a more realistic case the determination of the exact SF distances is more robust if the SF types are known *a priori*. The maxima in the Patterson function taken from nw2 (Fig. 10 ▸) do not allow for exact determination of SF distances. From the cross-sectional TEM image of nw2, it is evident that there are more SFs in comparison with nw1. This is the reason why less regular oscillations are observed in the speckle pattern in Fig. 3(*b*) ▸. In addition, another obvious reason why the direct determination of the fault positions from the Patterson function is not possible in nw2 is that by chance the differences 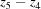 and 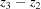 are quite close and the corresponding maxima in the Patterson function almost coincide.

Characterization of the number of SFs and distances in between them using the Patterson analysis described in this work shows that, despite the several constraints, namely low number of SFs and high sensitivity to noise, the approach is relatively fast and easy to apply in comparison with coherent diffraction imaging, where the data have to be analyzed by phase-retrieval or ptychographic techniques. Similar to other CXD experiments our technique required an isolated object and a sufficient coherent beam to allow for the observation of speckle patterns arising from the distance between individual defects. The method can be also used for other systems, where a small number of plane defects are observed in a well defined limited volume (microcrystals, grains *etc*.). The extreme sensitivity for the SF distances down to a single atomic plane can be used for a detailed investigation of the positions of planar defects in core/shell nanowires (Goldthorpe *et al.*, 2008 ▸), where SFs occur only in different positions in the shell structure and in the NW core.

## 7. Summary   

If a single wurtzite nanowire is irradiated by a fully coherent X-ray wave, the reciprocal-space distribution of the diffracted intensity exhibits oscillations whose periods depend on the mutual distances of stacking faults. In this paper we present an effective method of extracting the distances from these oscillations, based on the Fourier transformation of the diffracted intensity (Patterson function) calculated in a limited window in reciprocal space. The positions of the maxima of the Patterson function determine the distances between the SFs with an accuracy of about 10 nm. Moreover, the shape of the Patterson function sensitively depends on the fault plane distances so that from fitting the measured and simulated shapes of the Patterson function the distances can be determined with an accuracy of a single atomic plane. We have used this method for two GaAs nanowires; the resulting distances of the stacking faults exactly agree with the distances determined from transmission electron microscopy of the same nanowires.
